# Systemic Arterial Hypertension and Factors Associated with Blood Pressure Dysregulation in Companion Animals

**DOI:** 10.3390/vetsci12050453

**Published:** 2025-05-09

**Authors:** Felipe Gaia de Sousa, Fabiana Silva Fádel Queiroz, Ruthnéa Aparecida Lázaro Muzzi, Júlio César Cambraia Veado, Suzane Lilian Beier

**Affiliations:** 1 Department of Veterinary Clinic and Surgery, Veterinary School, Federal University of Minas Gerais—UFMG, Belo Horizonte 31620-295, MG, Brazil; fgaias@ufmg.br (F.G.d.S.); fabianafadel@ufmg.br (F.S.F.Q.); cambraia@ufmg.br (J.C.C.V.); 2 Department of Veterinary Medicine, Faculty of Animal Science and Veterinary Medicine, Federal University of Lavras—FZMV/UFLA, Lavras 37200-900, MG, Brazil; ralmuzzi@ufla.br

**Keywords:** behavioural factors, hypertension, haemodynamics, stress, white coat syndrome

## Abstract

Systemic arterial hypertension (SAH), characterised by a persistent increase in BP beyond the reference values for the species, is a concerning and detrimental clinical condition. The aim of this manuscript is to present the state of the art on SAH in companion animals, including the different types of hypertension, diagnostic and therapeutic approaches, and the factors associated with its occurrence, such as the role of stress. It also discusses the benefits and challenges related to the measurement process. SAH is categorised into types based on the underlying cause: situational, secondary, and idiopathic (the least frequently observed). The situational type occurs when stress is the primary factor, such as during veterinary visits, contact with other animals, or in cases of ‘white coat syndrome’. If the stressor is removed, BP values tend to normalise. The most common type of SAH is the secondary form, which is associated with an underlying condition, such as renal, cardiac, endocrine, or neurological diseases, or a combination of these. Diagnosing SAH is a challenging task for many veterinarians due to factors related to handling the animal, managing the equipment, and interpreting the results. Consequently, many professionals either take inaccurate measurements or misinterpret the results, often prescribing antihypertensive medications prematurely or unnecessarily. Despite being a well-standardised and well-documented process, challenges persist. Treatment often involves antihypertensive drugs, either alone or in combination, alongside management of the underlying causes, when present. Therefore, BP values should be obtained correctly, with clinical-therapeutic decisions carefully aligned with the factors that may influence them.

## Simple Summary

Cardiovascular diseases are characterised by clinical conditions that affect the heart and blood vessels, potentially leading to progressive and harmful alterations, whether local or systemic. Changes in cardiovascular haemodynamics can result in significant blood pressure (BP) variations, which, if poorly controlled, become detrimental to systemic health. Systemic arterial hypertension (SAH), characterised by progressive increases in BP, can be caused by various factors, including individual characteristics, environmental influences, and diagnostic methods. Failure to consider the multiple factors affecting BP can lead to false positive or negative diagnoses. Diagnosing hypertensive conditions is a challenge frequently reported by veterinary professionals, many of whom may unnecessarily or inappropriately prescribe pharmacological treatments. The BP measurement should be considered a routine clinical assessment method; however, it must be performed in a standardised and accurate manner, following well-defined criteria and accounting for factors such as stress and environment. It is known that this process is often overlooked or performed incorrectly. Therefore, the interpretation of BP values must take various associated factors into account, requiring caution in diagnostic determination.

## 1. Introduction

Cardiovascular diseases are clinical conditions that can be either primary or secondary to other events. Regardless of how they are characterised, they are routinely observed. The presence of cardiovascular disturbances or alterations is marked by significantly deleterious and/or harmful situations, which can cause discomfort for both the patient and their caregiver. These may include physical and mental fatigue, as well as a reduction in quality of life and life expectancy [1]. Generally, depending on the disease progression and other associated factors such as age, breed, and the type of condition, animals may frequently experience decompensated episodes, which should be effectively managed to ensure success in diagnostic and therapeutic management. New characteristics of urban centres, such as verticalisation, methods of animal husbandry, and emotional/behavioural support, contribute to increased vigilance from caregivers in observing clinical conditions that interfere with the animals’ lifestyle, as well as a greater dedication to health-related matters [1]. Among the various cardiovascular conditions and alterations, congenital diseases, acquired conditions, and those associated with BP are noted.

SAH is a condition characterised by the elevation of BP values above the reference values for the species, in a chronic manner. It can be classified into three types [1,2]. Frequently, most cases of SAH are associated with underlying causes such as renal, endocrine, or neoplastic diseases, among others. However, it is important to note that SAH can also be caused by situational factors, primarily induced by stress, as well as primary aspects (absence of underlying diseases). Stress is one of the main factors associated with situational hypertension, which is often observed in veterinary clinics and hospitals due to the occurrence of an exaggerated sympathetic discharge. When animals are exposed to unfamiliar situations, such as new environments, the presence of other dogs and/or cats, and various odours, the release of circulating catecholamines can occur, maintaining the sympathetic system in a state of alert [3]. Therefore, the clinical evaluation of animals in a state of stress may be under- or over-influenced, meaning that the variables collected may not reflect the true condition of the animal. It is expected that BP values in dogs influenced by sympathetic activation will be higher when compared to patients in calmer environments. Thus, it is essential to consider stress during BP measurement to minimise its effects as much as possible. According to Sousa et al. [1], “hypertension often presents silently, and as such, its identification by caregivers becomes imperceptible, as animals remain asymptomatic”. Diagnosing hypertension can be challenging, especially due to “white coat syndrome” (elevation in BP triggered by the presence of professionals dressed in white coats). The diagnosis of hypertension can be established when elevated BP values are consistently observed in a standardised manner, with multiple measurements taken at different times [2]. The treatment varies and depends, in part, on the underlying causal factor. Antihypertensive medications may be prescribed, either alone or in combination. The aim of this manuscript is to explore SAH in companion animals, outlining its types, diagnostic and therapeutic approaches, associated factors such as stress, and the benefits and challenges of accurate and well-executed measurement.

## 2. Blood Pressure

The measurement of BP has taken on an important role in veterinary medicine, and the recognition of SAH in dogs in recent years underscores the importance of this measurement being performed in a systematic manner. Notably, understanding BP and its disorders in companion animals, as well as the technology for its measurement, has evolved over time [2]. The measurement of BP in dogs and cats is a method derived from human medicine, with the first recorded BP measurement taking place in 1733 by Stephen Hales using an invasive method (later described) in a mare. Later, around 1895, the Italian Riva-Rocci determined BP through digital palpation. The significant achievement of the auscultatory method was made by the Russian Korotkoff, crowning medicine with an examination of great importance and implications in various diseases [4,5,6]. Given the prominence of BP evaluation, several studies and guidelines similar to those developed for humans have been conducted and published in veterinary medicine, such as the Consensus Statement of the American College of Veterinary Internal Medicine (ACVIM). Moreover, there is a strong emphasis on veterinarians using BP measurement as a requirement to be evaluated during medical consultations, given that more than 80% of hypertensive cases are secondary to other diseases [7]. Consequently, understanding BP and its consequences in companion animals becomes fundamental in attempting to predict the potential developments of elevated BP.

Cardiovascular function is based on central strategies with the ultimate goal of ensuring adequate blood volume supply that can promote proper cardiac pumping, oxygenation, nutrition, and tissue and/or organ perfusion [8,9,10]. For this, intrinsic and extrinsic regulatory processes are required. For proper cardiac pumping, there must be cardiac output (CO) and venous return (VR); however, depending on the present condition, haemodynamic variations may occur [9]. Such modifications are mediated by need, so that the greater the volumetric demand (e.g., intense physical exercise), the greater the amount of blood supplied [8]. Situations involving variability in preload and afterload values may lead to haemodynamic modifications, with the potential for organ depletion and/or damage [8].

BP dysregulation is swiftly controlled by the activation of compensation mechanisms, whether acute or chronic, aimed at controlling or halting the causal process. It is important to emphasise that although compensatory, cardiovascular afflictions are progressive, which can aggravate the problem through hyperactivation of regulatory mechanisms, leading to harmful and detrimental processes [8,9,10]. Several haemodynamic regulatory processes are described, such as the Frank–Starling and Laplace laws, the sympathetic nervous system (SNS), and the activation of the renin–angiotensin system (RAS), as well as acute or long-term local controls (e.g., vasodilatory theory, oxygen demand), among others [8,9]. Regardless of the type of regulation, whether isolated or not, the central goal is to regulate and maintain haemodynamic status to avoid temporary and/or permanent damage and loss [8,9].

In this context, the BP values of dogs and cats are variable, and various factors, such as the skill of the operator performing the measurement, the method employed, the behaviour of the animals, and the choice of cuff, can interfere with the results [2]. Therefore, standardising the measurement process to reduce measurement bias is strongly recommended [2]. Other variables such as the animal’s size, breed, sex, body condition, and environment can also cause BP variability [2,11]. In humans, age is a variable that affects arterial structure, which could explain why elderly individuals tend to have higher BP values [12]. According to Acierno et al. [2], the effect of age on the BP of dogs remains under-studied. Bodey and Michell [13] found evidence of an annual increase in BP of approximately 1–3 mmHg. This phenomenon was also described by Bodey and Sanson [14], Bright and Dentino [15], and Payne et al. [16]. Bijsmans et al. [17] conducted an experiment with senior cats to evaluate if BP increased with age and observed that BP values were higher in felines over 9 years old, particularly in those with underlying disease.

Regarding sex, there are still gaps in fully understanding the influence of sex on BP values [13,18,19]. Bodey and Michell [13], after analysing more than 1900 dogs, found that sex influenced BP, with lower BP observed more strongly in females. It is important to note that the difference in BP between neutered and intact individuals was 10 mmHg [13]. According to Bodey and Sansom [14] and Mishina et al. [20], the influence of sex is not well-established in studies because many studies work with neutered animals, reducing the influence of sex hormones. Payne et al. [16] found that, in cats, the average BP was higher in males compared to females (122.2 mmHg vs. 119.6 mmHg), with the increase being more pronounced in neutered cats. Breed is strongly associated with BP, with Acierno et al. [2] suggesting that BP should be described by breed. Some studies have shown that Greyhounds and Great Danes have BP values 10–20 mmHg higher than mixed-breed dogs [13,18]. Bodey and Rampling [21] highlighted that hunting dogs had higher BP values than retriever breeds. However, according to Payne et al. [16], the racial component is still not well-defined. For Bodey and Michell [13] and Rattez et al. [22], BP can also vary with temperature values, with fluctuations of around 7–10 mmHg. However, most studies do not assess the influence of temperature on BP. According to Bodey and Mitchell [13], “systolic BP is the most variable pressure parameter and depends on age, breed, sex, temperament, disease status, exercise regimen, and, to a lesser extent, diet”, indicating that BP variability is influenced by numerous causes. The relationship between obesity and BP in SAH has been demonstrated in various studies [23,24]. According to Mendes et al. [25], “obesity is associated with chronic cardiac volume overload, increased cardiac output, and activation of the renin–angiotensin–aldosterone system (RAAS) and the sympathetic nervous system (SNS)”.

The influence of obesity on BP is often associated with underlying cardiovascular repercussions [19,26]. In the study by Pereira-Neto et al. [26], the authors observed that the presence of adipose tissue influenced BP measurements, as the vascular method showed differences compared to the oscillometric method in obese dogs. In the study by Payne et al. [16], the “body condition” factor was correlated with BP values, with cats scoring below ideal (4/9) showing lower BP values compared to those with scores of 5 or higher. Rondeau et al. [27] and Mooney et al. [28] described that sarcopenic processes and muscle evaluation can lead to variability in BP values, especially if measured in the radial artery. In general, Acierno et al. [2] emphasise that “blood pressure measurement results in normal animals are highly variable based on breed, temperament, patient position, measurement method, operator experience, and intra-patient variability, making it difficult to determine a single value or range that applies to all dogs or cats”.

## 3. Systemic Blood Pressure Regulation Systems

The BP can be regulated locally and/or systemically through the activation of compensatory and regulatory systems [10]. Pressure compensation is based on physiological strategies, both short-term and long-term, aimed at stabilising the body in response to the newly imposed condition. Intrinsic and extrinsic mechanisms may be activated, either independently or together, to support the organ system and prevent the onset or worsening of secondary conditions resulting from pressure fluctuations, such as hypoxia and tissue/organ ischaemia [8].

### 3.1. Sympathetic Nervous System

**Figure 1:**
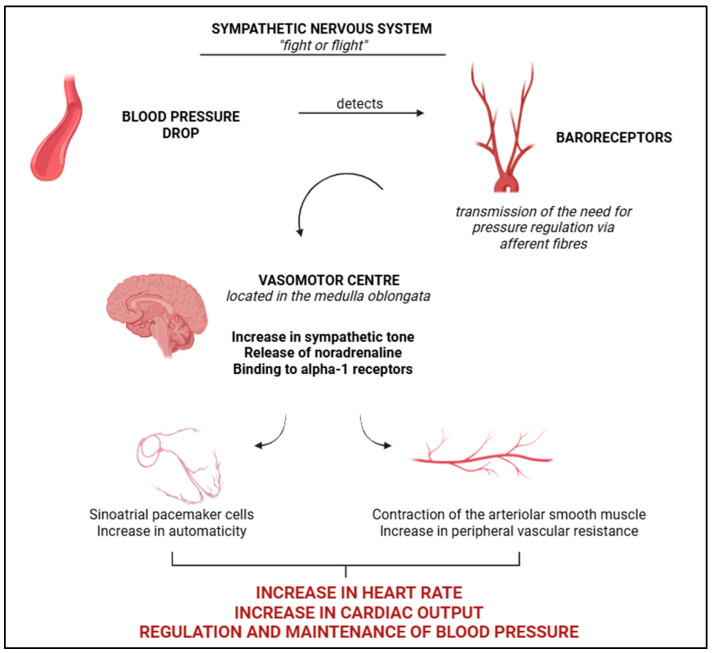
Schematic representation of the SNS, derived from the autonomic nervous system, to ensure haemodynamic stabilisation, to increase heart rate, cardiac output and regulation/maintenance of blood pressure. BioRender 2025 (https://www.biorender.com/ (accessed on 10 January 2025)). Created by the authors.

The SNS, along with the parasympathetic nervous system (PNS), forms the autonomic nervous system, with the former exerting a more significant regulatory effect on BP. Neural control of BP is governed by the nervous system, which regulates blood flow, pressure, and cardiac function, alongside the presence of sympathetic fibres that induce changes in volumetric and peripheral vascular resistance (PVR) (Figure 1) [10]. Primary regulation via the SNS is associated with a greater number of sympathetic fibres, resulting in heightened responsiveness of various parts of the body, such as the kidneys, skin, and intestines, to norepinephrine [29]. The system is activated in response to a drop in BP and changes in the distensibility of blood vessels, conditions promptly detected by baroreceptors, which transmit information via afferent pathways to the brainstem (vasomotor centre), facilitating the release of norepinephrine and its binding to alpha-1 receptors (Figure 1) [29]. Following this process, the smooth muscle layers of the arteries contract, increasing PVR. Additionally, there is an enhancement in the automaticity of the sinoatrial node, resulting in an increased heart rate, cardiac output, stabilisation of vascular resistance, and the re-establishment of haemodynamic balance (Figure 1) [29]. However, while these haemodynamic adjustments are initially beneficial, as the system becomes hyperactivated, there is impairment in the relaxation and volumetric accommodation phases, leading to cardiac cycles with lower cardiac output and reduced perfusion [9,10]. In this regard, Sousa [10] emphasises that the system ceases to be beneficial and, over the long term, becomes detrimental.

### 3.2. Renin Angiotensin System

**Figure 2:**
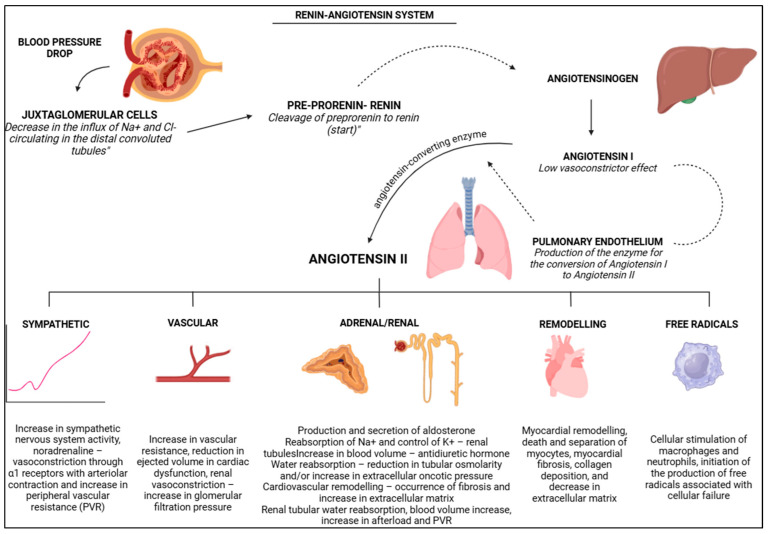
Schematic representation of the renin–angiotensin system (SRA), highlighting the role of the kidneys in haemodynamic stabilisation, with effects in sympathetic system, vascular and adrenal/renal modifications, remodelling and production of free radicals. BioRender 2025 (https://www.biorender.com/ (accessed on 10 January 2025)). Created by the authors.

The RAS can be activated locally and/or systemically, playing a key role in compensating for haemodynamic changes, particularly through renal function (Figure 2) [10]. The function of the RAS is based on BP regulation [30,31,32,33], PVR regulation [34,35], and the maintenance of hydroelectrolytic balance [31,36,37]. Activation of the RAS is triggered by conditions related to organic, tissue, and haemodynamic disturbances [38]. RAS compensation can be acute and/or chronic; however, most compensation arises from chronic activation [32,34]. According to Sousa [10], compensation is initially beneficial to organic control; however, over time, as the condition progresses, the RAS becomes a detrimental and harmful system. McKinney et al. [31] argue that the system becomes damaging when there is persistent tissue damage and/or injury, along with hyperfunction and hyperactivity of the cardiorenal system, as well as organic imbalance. As a pleiotropic system, compensation occurs through the reduction in BP and alterations in water and electrolyte availability, with the kidneys (macula densa) detecting these changes (Figure 2) [10,39,40]. According to Vargas et al. [41], the juxtaglomerular cells, located in the middle of the afferent arterioles [36], detect the decrease in circulating sodium and chloride ions in the distal convoluted tubule [37], which triggers the conversion of prorenin to renin [34,36,42]. It is renin that initiates the entire process via the RAS after stimulation (Figure 2) [40].

Renin facilitates the breakdown of angiotensinogen into angiotensin I (which has low vasoconstrictor activity) (Figure 2) [10,33,34,35,38,40,42,43,44,45,46]. Angiotensin I is quickly converted into angiotensin II by the angiotensin-converting enzyme (ACE) [38]. Once formed, angiotensin II plays roles in BP control, cardiac adjustment, haemodynamic regulation, and electrolyte balance [36]. Much of the effects of angiotensin II are variable and associated with vascular, nervous, and hormonal actions, cardiovascular remodelling, as well as free radicals and reactive oxygen species (Figure 2) [10]. It is emphasised that, according to Sousa [10], the effects mediated by angiotensin II are initially beneficial; however, as the condition worsens, clinical changes become evident to organic balance. Figure 2 summarises the RAS.

## 4. Systemic Arterial Hypertension

SAH is a clinical condition characterised by persistently elevated BP values in relation to the reference parameters for the species [2]. However, according to Sousa et al. [1], the diagnosis of SAH should be based on certain factors that could interfere with obtaining reliable values. Therefore, the diagnosis should be based on measurements that are in accordance with the environment and the behaviour of the animal, “in order to avoid underestimating or overestimating blood pressure values” [1]. Currently, conditions of measurement, waiting for clinical attention, patient management, interaction with other animals and/or species during pre-consultation periods, transportation, and acclimatisation to the environment are factors that influence BP [1]. In this context, it is emphasised that the identification of SAH should be approached with caution, supported by clinical evidence, and in accordance with the animal’s environment and behaviour, as pharmacological therapy should only be administered after the diagnosis.

According to Acierno et al. [2], SAH can be subclassified into three types: situational, idiopathic, and secondary. The categorisation process was based on the presence of situations that led to BP changes but which could have another underlying cause, such as stress, conditions that cause vascular and haemodynamic alterations, or periods when potential causes were ruled out, and BP values remained elevated [1]. It should be noted that many professionals, unfortunately, still make incorrect diagnoses of SAH due to factors such as errors in the measurement process, single and isolated measurements, or failure to consider influencing factors. As a result, the existence of inaccurate diagnoses affects clinical and therapeutic management.

### 4.1. Situational Arterial Hypertension

**Figure 3:**
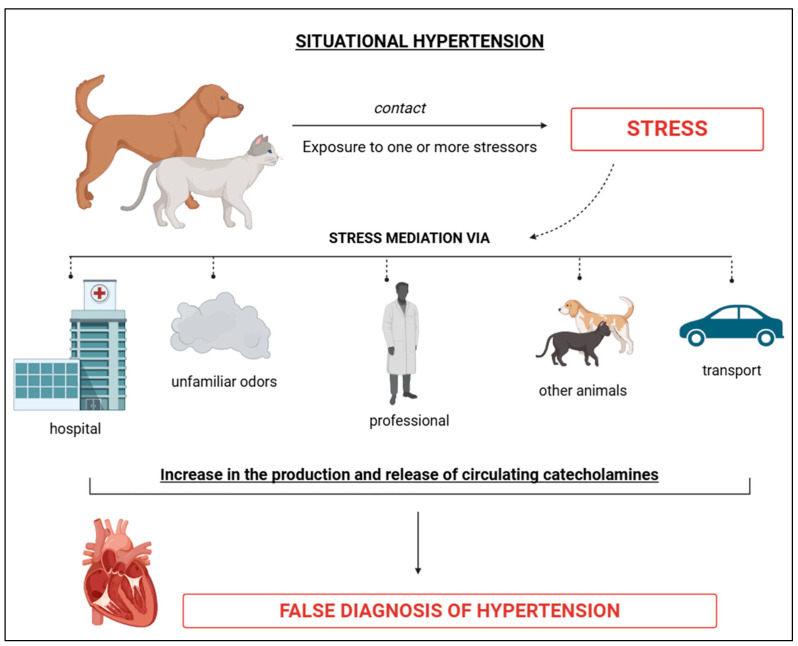
Schematic representation of situational arterial hypertension, characterised by the presence of an event frequently associated with stress factors, temporary, capable of elevating BP values (false diagnosis of hypertension). BioRender 2025 (https://www.biorender.com/ (accessed on 10 January 2025)). Created by the authors.

Situational SAH is characterised by a temporary or momentary increase in BP, often triggered by the presence of one or more stress-inducing factors (Figure 3) [1,47]. According to Acierno et al. [2], it is “caused by alterations in the autonomic nervous system resulting from the effects of arousal or anxiety on the higher centres of the central nervous system”. Sousa et al. [1] state that this form of hypertension is frequently observed in veterinary clinics and hospitals is associated with the “white coat syndrome” (Figure 3). Data suggest that the sight of individuals wearing white attire can induce a certain repulsion, which may explain the occasional increases in BP. Situational hypertension tends to resolve once the causal stressors are controlled or eliminated, reducing or eliminating physiological stimuli with an anxiolytic effect [2]. Since situational hypertension is temporary and caused by stress without underlying issues, therapeutic management is not required (Figure 3). Instead, the focus should be on monitoring and re-measuring after the stressor is removed or controlled [2].

As noted by Lyberg et al. [11], factors such as veterinary environments, the process of acclimatisation in transport boxes, transportation, waiting, and return to the home environment can contribute to temporary increases in BP values. The auditory and olfactory influence of animals’ exposure in veterinary clinics and hospitals should also be emphasised (Figure 3). It is notable that BP measurements can exhibit significant variability depending on the environment in which they are taken (Figure 3). The unfamiliar environment and the physical restraint for clinical and supplementary assessments can affect temporary hypertension, potentially leading to underestimation or overestimation [1,11] (Figure 3). This environmental influence may also explain why BP measurements are typically lower at home compared to veterinary offices or hospitals, as the home environment is more familiar and comfortable (Figure 3). Given these findings, it is essential to consider the environmental variable, whether associated with stress or not, before making diagnostic and therapeutic decisions regarding hypertension.

### 4.2. Idiopathic Arterial Hypertension

**Figure 4:**
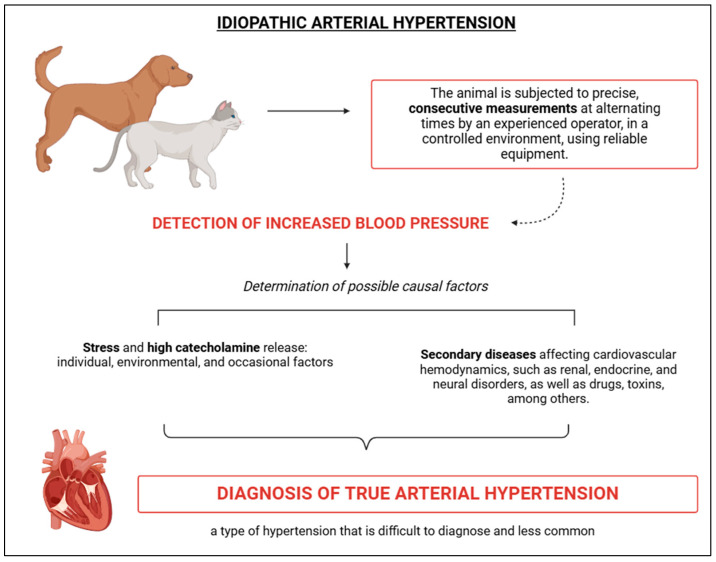
Schematic representation of idiopathic arterial hypertension, characterised by elevated blood pressure values in the absence of stress or secondary diseases, described as true hypertensive condition (less common diagnosis). BioRender 2025 (https://www.biorender.com/ (accessed on 10 January 2025)). Created by the authors.

Idiopathic SAH, or true hypertension, is characterised by a persistent increase in BP values, provided that contributing factors such as stress and secondary diseases are ruled out (Figure 4). Idiopathic hypertension is associated with intrinsic and/or extrinsic variables, lacks an underlying cause, and, given that a significant number of animals present with clinical conditions that interfere with their haemodynamic status, its diagnosis can be challenging [2]. Moreover, according to Acierno et al. [2], “the presence of chronic hypertension suggests that one or more of the neuro-humoral and renal systems responsible for regulating blood pressure are abnormal”, once again highlighting the influence of the cardiovascular–renal–neuroendocrine axis in the occurrence of hypertension (Figure 4). The diagnosis of true hypertension focuses on excluding all potential causes, with the aid of diagnostic tests that rule out hepatic, renal, endocrine, and other alterations (Figure 4) [2]. According to Acierno et al. [2], idiopathic SAH should be suspected when reliable blood pressure data are obtained in conjunction with laboratory profiles showing values within the normal range (complete blood count, serum biochemistry, and urinalysis). Furthermore, the authors highlight that elevated blood pressure may lead to polyuria. Therefore, animals presenting with urine specific gravity values below 1.030, alongside increased blood pressure, should not be diagnosed with chronic kidney disease (CKD); likewise, a high specific gravity also rules out the presence of CKD. As such, Acierno et al. [2] recommend that additional tests be conducted to confirm or exclude secondary causes of SAH, such as ultrasonography, renal biomarkers, and measurements of thyroxine, cortisol, aldosterone, among others. Specifically for felines, approximately 13–20% of these cases are idiopathic hypertension [48,49,50].

### 4.3. Secondary Arterial Hypertension

**Figure 5:**
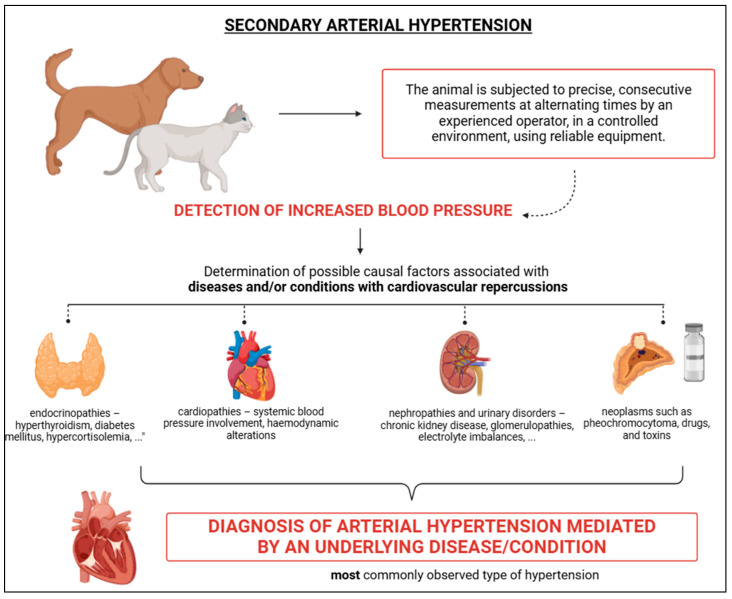
Schematic representation of secondary arterial hypertension, characterised by elevated blood pressure values resulting from an underlying cause such as nephropathies, endocrinopathies (more common), among others. It is considered the most common type of hypertension. BioRender 2025 (https://www.biorender.com/ (accessed on 10 January 2025)). Created by the authors.

The most frequently diagnosed form of SAH is secondary hypertension, which arises from an underlying cause, typically linked to a specific clinical condition. According to Acierno et al. [2], this condition is characterised by a consistent increase in BP resulting from a baseline condition that is sufficiently capable of elevating BP values (Figure 5). It is important to highlight the role of certain medications (iatrogenic) and neoplasms in this form of hypertension [2]. Such increases in BP, caused by an underlying condition, are more commonly observed in middle-aged and geriatric patients, as diseases related to hypertensive phenomena, such as CKD, endocrinopathies, congestive heart failure or other cardiopathies, are frequently seen (Figure 5) [2]. Often, secondary hypertension presents as clinical symptoms rather than as an isolated clinical condition. Even though therapeutic management of the underlying cause may be implemented, BP control may not always be effective. In some cases, BP values may not decrease, may return to reference levels, or may even increase [51,52]. Due to the presence of an underlying cause, continuous clinical and laboratory monitoring is necessary. A study by Sparkes et al. [53] confirmed the influence of underlying conditions in the onset of hypertension, particularly the concomitant presence of CKD and/or hyperthyroidism (Figure 5).

## 5. Stress and Catecholamine Release

Stress is a physiological response of the organism to situations that induce overactivation and the release of circulating catecholamines. These stress responses can be triggered by various conditions, such as blood sample collection, fight-or-flight situations, and conflicts with other animals. Since 1998, Broom [54] has defined stress as an organic reflex triggered by the perception of an event considered an obstacle to the animal’s equilibrium, initiating both behavioural and physiological changes to confront the stressor. Lucassen et al. [3] further describe stress, noting that aversive, unpleasant, or threatening situations activate the organism in response. According to Mârza et al. [55], stress is often linked to environmental factors, where poor environmental enrichment, spatial restrictions, and limited sensory and cognitive stimulation are common stressors. Broom [54] and Mârza et al. [55] highlight that stress can be acute or chronic, with continuous hyperactivation and hypervigilance being more harmful than transient, short-lived situations. Thus, while stress is a physiological response to an inciting event, its impact can have significant detrimental effects.

Lucassen et al. [3] describe stress as mediated by two responses: the alarm phase and the glucocorticoid-regulated phase. The first phase, the alarm phase, is based on the fight-or-flight response mediated by the SNS, which leads to the release of epinephrine and norepinephrine. These hormones trigger organic changes such as increased metabolism, BP, and respiration, as well as heightened vasoconstriction, which redirects blood flow to vital organs like the brain and muscles. The second phase involves the release of glucocorticoids (e.g., cortisol), which help the organism adapt to the stressor by influencing metabolic variables [3].

Stress in veterinary medicine has become an area of growing concern, especially as pet owners increasingly focus on their animals’ well-being. Companion animals, for example, cats and dogs, face multiple stressors in various situations, including veterinary evaluations. Especially in cats, stress components are more commonly observed, particularly in relation to containment and handling processes. This justifies the use and need for Cat Friendly practices to ensure a lesser negative impact during veterinary evaluations. Additionally, animals subjected to continuous stress, such as those in poor living conditions (e.g., chained, confined to small kennels with poor hygiene, or lacking socialisation), are also notably affected. In veterinary settings, stress can complicate or even prevent the proper conduct of clinical and supplementary examinations, leading to potential diagnostic and therapeutic errors. Lee et al. [56] found that environments with ample space, socialisation opportunities, and environmental enrichment strategies were associated with reduced stress.

Research by Vincent and Michell [57] showed that animals prone to stress could exhibit elevated BP levels. Stress is a significant factor, particularly when associated with “white coat syndrome”, where physiological responses are triggered in dogs upon encountering the white coat, due to fear, aversion, or hypervigilance (Figure 3). This suggests that animals under stress are experiencing momentary organ hyperfunction, making BP measurement in these situations inadvisable. Acierno et al. [2] recommend that BP measurements be taken after a 10–15 min acclimatisation period to allow stressors and circulating catecholamine levels to decrease. In human medicine, the phenomenon of “masked hypertension” may occur, where a patient’s BP readings decrease upon entering a hospital setting [58,59]. Given this possibility, home BP monitoring has been considered a complementary or auxiliary assessment method [58,59]. However, this period may sometimes be insufficient for these changes to occur. In some cases, BP measurements at home, where animals are in a familiar environment, can provide more accurate readings [2].

Studies highlight the variability in BP readings between different environments, with values generally lower at home. Soares et al. [60] compared BP readings in 45 dogs and found the mean BP at home was 136.3 mmHg, while in the veterinary office it was 154.7 mmHg, with the environmental variable being the most likely cause of the difference. Preliminary results from Queiroz et al. [61] indicated that the mean hospital BP was 189.5 mmHg, while home readings were within the species’ reference range (138 mmHg). Queiroz et al. [61] attributed this BP variability to stress, suggesting that environmental factors contribute to the increase in values. Koo and Carr [62] also found that both systolic and diastolic BP were higher in a veterinary office, likely due to “white coat syndrome”. Identifying the specific stressor responsible for the changes is crucial, as each animal responds differently, making it challenging to pinpoint the exact cause. Sousa et al. [1] emphasise that many professionals are unaware of how catecholamines and stressful events influence cardiovascular hemodynamics. This lack of understanding could lead to unnecessary prescriptions of antihypertensive drugs.

## 6. Laboratory Biomarkers of Stress

### 6.1. Cortisol

**Figure 6:**
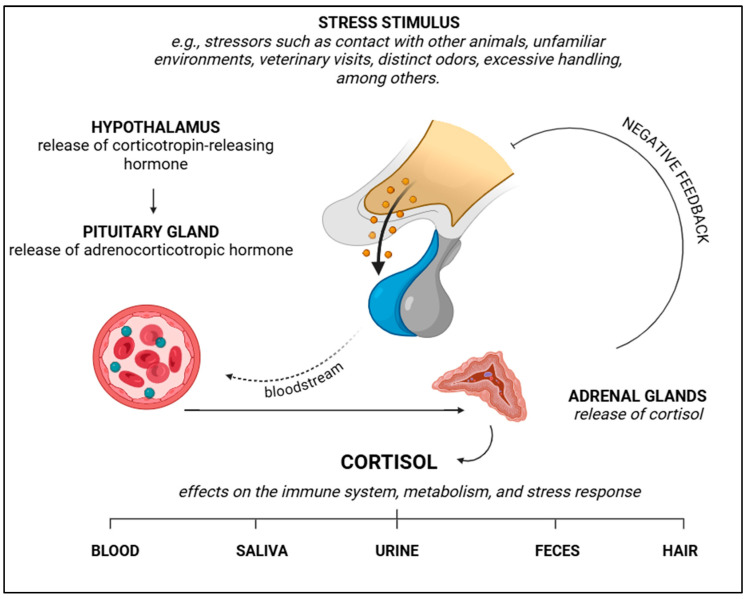
Schematic representation of cortisol release through the hypothalamic–pituitary–adrenal axis, after stimulus, as well as the samples in which the hormone can be analysed. BioRender 2025 (https://www.biorender.com/ (accessed on 10 January 2025)). Created by the authors.

In addition to catecholamines, which play a crucial role in stress development, cortisol is another important factor. Cortisol is a glucocorticoid produced by the adrenal glands, and it is involved in the body’s regulatory processes, playing a key role in the stress response (Figure 6) [55]. The role of cortisol in veterinary practice gained prominence due to its impact on endocrine and behavioural disorders, and it is often considered a biomarker of stress [55,63]. However, it is important to note that elevated serum cortisol levels do not necessarily lead to negative outcomes, especially in animals for whom alertness is essential, such as hunting dogs or guide dogs for the visually impaired [55,64].

Cortisol is produced in the fasciculata region of the adrenal glands in response to corticotropin-releasing hormone and adrenocorticotropic hormone (Figure 6) [65,66]. The presence of adrenocorticotropic hormone stimulates cortisol production and release into the bloodstream, which is necessary for dogs to manage stress and regulate metabolic and immune functions (Figure 6) [55]. Cortisol levels can be influenced by various factors, including the environment, physiological states, and behaviour, making its regulation a complex process [55,63].

Cortisol is regulated by a feedback loop involving the hypothalamic–pituitary–adrenal axis, which is crucial for the body’s adaptation to stress, both acutely and chronically (Figure 6) [8,55]. Environmental factors also influence cortisol levels, with positive interactions and the presence of owners helping to reduce circulating cortisol levels, while the opposite can be true as well [55]. Petterson et al. [67] suggest that cortisol levels in both owners and animals can be influenced by how they interact and respond to each other. It is also important to note that cortisol levels can fluctuate throughout the day, particularly in high-competition environments, such as when animals compete for space, food, or during testing [55,63] (Figure 6).

From a behavioural perspective, cortisol measurements are valuable for assessing the environment in which animals are placed, as well as the strategies for veterinary assessment and management. It can also help monitor strategies aimed at stress reduction [55,63]. Mârza et al. [55] discuss how individuals may exhibit behavioural differences, which can be categorised as the “shy versus bold behavioural syndrome”. “Bold” animals tend to be more exploratory and risk-prone, while “shy” animals are more cautious and risk-averse. These differences are linked to cortisol responses, with “bold” animals showing larger increases in cortisol levels (due to lower baseline levels), whereas “shy” animals show smaller increases due to higher baseline levels. The behavioural characteristics of each animal may influence their response to stress and coping mechanisms. Furthermore, Mârza et al. [55] explain that cortisol regulation is influenced by both environmental and social factors, and dysregulation can affect stress dynamics and overall physiological functioning.

Cortisol levels can be measured using various methods, such as blood, saliva, urine, hair, and faecal samples (Figure 6). For stress assessment, cortisol is a prominent biomarker, as it reflects both stress levels and overall well-being [55]. Rosado et al. [68] highlight that elevated cortisol levels can also be linked to territorial dominance. Each method of cortisol measurement has its specific advantages and challenges. Blood sampling is an invasive technique that may cause temporary stress following collection, but it provides definitive results [69,70]. Saliva samples are less invasive and can offer insights into acute stress levels; however, the values may be influenced by dietary components and the type of collection material used [55,71,72].

Urine samples can also be used to measure cortisol, though they are more useful for detecting chronic stress [55]. The cortisol-to-creatinine ratio in urine is particularly valuable for evaluating cortisol, especially in endocrine conditions [55,73]. However, urinary cortisol measurements are more commonly used for assessing chronic stress rather than acute stress [55]. It is worth noting that cortisol measurements in urine can vary by breed; for example, Akita dogs tend to have higher cortisol levels compared to Labrador Retrievers, as shown by Nagasawa et al. [74]. For chronic stress evaluations, hair and faecal samples are also useful [75]. The choice of method depends on the context and the type of stress being evaluated.

### 6.2. Glucose

Laboratory changes in glucose levels are commonly observed during stressful situations. According to Hagley et al. [76], episodes of hyperglycaemia can occur as a result of stress, even in the absence of underlying diseases. Magomedova and Cummins [77] explain that the increase in serum glucose due to stress is part of an organic response, where the central nervous system stimulates the hypothalamic–pituitary–adrenal axis. Additionally, Liu et al. [78] describe an increase in serum glucose mediated by the hypothalamic–sympathetic–liver axis, without necessarily involving adrenal function during stress, as shown in a murine model. This mechanism is known as “stress-induced hyperglycaemia” [76], and it is frequently observed in cats [79,80].

The “stress-induced hyperglycemia” mechanism, as described by Rand et al. [79], leads to a transient increase in glucose levels, which can sometimes cause confusion in the diagnosis of diabetes mellitus. Liu et al. [78] explain that, during acute stress, the body releases glucose into the bloodstream to ensure an energy reserve. This excessive release of blood glucose is understood in the context of survival mechanisms between predator and prey [78]. In such predatory situations, the vulnerable prey must have higher glucose levels to fuel an escape response. Liu et al. [78] suggest that “stress-induced hyperglycaemia” occurs in stages: the rapid stage (around 3 min), driven by the hypothalamic–sympathetic–liver axis; the delayed stage (3–30 min), mediated by the adrenal glands; and the late stage (15 min), influenced by the hypothalamic–pituitary–adrenal axis, completing within 45 min.

Thus, the occurrence of hyperglycaemia is linked to the presence of corticosteroids in the bloodstream, which promote gluconeogenesis and the release of glucagon [81]. This process causes insulin resistance, meaning that insulin is unable to regulate blood glucose levels, allowing for a transient accumulation of glucose [81]. Certain stress-inducing situations in dogs, such as those caused by heat, may also lead to changes in glucose levels. Azeez et al. [82] observed that in environments with high temperature and relative humidity, glucose levels were lower compared to environments with lower humidity, which also affected cortisol levels. While the pathway of hyperglycaemia during acute stress is well-documented, few studies have focused on this relationship in veterinary patients.

### 6.3. Stress Leukogram

The stress leukogram is a laboratory condition that occurs in animals experiencing high levels of stress, characterised by changes in haematological components such as neutrophils, eosinophils, lymphocytes, and monocytes [83]. This condition is typically observed in animals under stress, often in situations that complicate sample collection, such as restraint or repeated blood draws. While the stress leukogram is more commonly seen in felines due to their inherent stress sensitivity [84], it can also occur in dogs and influence diagnostic and therapeutic decisions.

Physiological stress, a specific type of stress, is associated with the production of adrenocorticotropic hormone, release of cortisol and can affect various cellular groups, including lymphocytes, neutrophils, monocytes, and eosinophils [83]. The most common alteration in a stress leukogram is lymphopenia, which results from lymphocyte death, along with a shift in cell populations [83]. Additionally, neutrophilia may occur, manifesting as an increase in neutrophil numbers without a shift, with the neutrophils often appearing hypersegmented [83]. Eosinopenia is another common feature, due to the influence of cortisol, and monocytosis can also be observed [83]. Although platelet aggregation is not recognised as part of the stress leukogram, it can still provide valuable insight into the animal’s stress levels during sample collection. A study by Benjamin et al. [85] showed a reduction in platelet aggregates in patients following the administration of trazodone, suggesting that pharmacological tranquillisers can mitigate platelet alterations caused by stress.

## 7. Diseases Associated with SAH

SAH can be associated with a variety of underlying conditions, and it is crucial to confirm or rule out these causes before making a definitive diagnosis of elevated BP. These conditions often have different levels of cardiovascular repercussions, which can lead to clinical alterations in various physiological aspects. Hemodynamic changes, the effects of urinary conditions linked to the heart–kidney axis, hormonal influences, and toxicity from harmful substances are all factors that can contribute to cardiovascular changes, potentially triggering hypertension. Therefore, it is essential to suspect and thoroughly investigate the presence of these conditions.

### 7.1. Chronic Kidney Disease and Urinary Disorders

The CKD and its associated urinary manifestations are primary underlying causes of hypertension [86], characterised by both functional and structural kidney failure. According to Sousa et al. [1], CKD is marked by a “failure in the excretory, regulatory, and endocrine functions of the kidneys”. Lawson and Jepson [87] define the diagnosis of CKD as the presence of renal alterations lasting for more than three consecutive months. Animals affected by CKD often experience significant morphofunctional changes, including the replacement of nephric tissue with fibrous mass, which impairs renal function [1]. Several conditions associated with CKD, such as alterations in sodium concentrations, glomerular filtration rate (GFR), glomerular hypertension, supernephrons leading to higher filtration rates, and renal sclerosis, contribute to the development of hypertension [1]. Notably, these conditions are frequently accompanied by proteinuria, which can vary in severity depending on the stage of CKD [88]. Moreover, the influence of the SNS, RAS, vascular changes, and reduced levels of vasodilators play key roles in the pathophysiology of both CKD and hypertension [87,88,89,90]. Hypertension is commonly observed in patients with CKD [53].

### 7.2. Endocrine Disorders

Endocrine diseases such as hyperthyroidism, diabetes mellitus, hyperaldosteronism, and hyperadrenocorticism are conditions frequently associated with BP alterations (Figure 5) [1]. Hyperthyroidism, characterised by excessive production of thyroid hormones, is more commonly observed in felines [91]. Thyroid hormones are believed to influence inotropic and chronotropic actions [92], with increased sensitivity of catecholamines to adrenergic receptors. This leads to an elevation in heart rate and contractility [93]. Williams et al. [94] note that hypertension in hyperthyroid animals is often linked to associated conditions rather than the disease itself, though BP tends to improve following treatment [95].

Diabetes mellitus, a complex and multifactorial condition characterised by impaired insulin production and/or action, is also associated with hypertension (Figure 5) [96]. Reusch et al. [7] point out that the origin of hypertension in diabetes is not fully understood, although reduced vasodilation due to insulin deficiency is suggested as a potential cause. Acierno et al. [2] report that hypertension occurs in 35–46% of diabetic cases. Hyperaldosteronism, which involves excessive aldosterone production by the adrenal glands, leads to hypertension through hydroelectrolytic and haemodynamic alterations, such as increased sodium retention and PVR [7].

The primary condition causing hypertension in animals is hyperadrenocorticism, which is characterised by excessive glucocorticoid (mainly cortisol) production and is most commonly seen in dogs (Figure 5) [97,98,99,100]. In cases where hyperadrenocorticism is ACTH-dependent, hypertension is observed in 86% of untreated cases, and even when treated, hypertension may remain persistent [52,101]. Goy-Thollot et al. [52] suggest that elevated cortisol levels may influence the vasculature, contributing to hypertension. Chen et al. [102] found that BP in dogs with hyperadrenocorticism was higher than in control groups. Evidence points to superactivation of the RAS and increased vascular sensitivity to angiotensin II, contributing to BP variability in these dogs [52,101], although further research is needed (Figure 2) [93].

### 7.3. Neoplastic Causes, Drugs and Toxins

SAH can also be mediated by factors such as neoplasms, drugs, and toxins, making it crucial to investigate the use of these substances (Figure 5). One example is pheochromocytoma, a neoplasm of chromaffin cells in the adrenal glands, which leads to increased production and concentration of catecholamines [86,93]. In these cases, hyperactivation of adrenergic receptors often occurs, resulting in hypertension [86]. The overstimulation of adrenergic pathways can lead to increases in heart rate, contractile force, and PVR, all of which contribute to elevated BP [93]. Additionally, certain drugs and toxins, such as glucocorticoids, mineralocorticoids, erythropoietin stimulants, phenylephrine hydrochloride, ephedrine, toracenib, cocaine, and amphetamines, can also be contributing factors to hypertension [2].

## 8. Target Organ Damage

**Figure 7:**
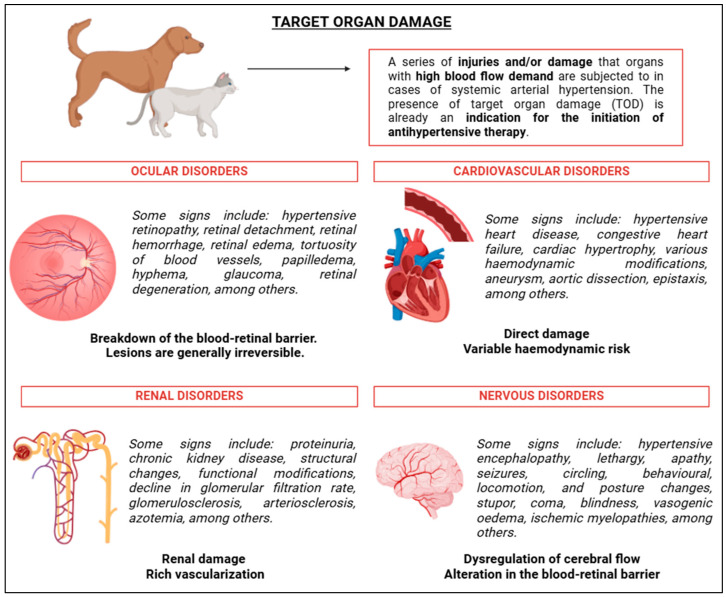
Schematic representation of target organ damage, including the organs and the most commonly found lesions in the presence of hypertensive conditions. BioRender 2025 (https://www.biorender.com/ (accessed on 10 January 2025)). Created by the authors.

Target organ lesions (TOD) are common manifestations observed in patients with SAH [87]. A significant number of animals diagnosed with SAH are at high risk of developing TOD during their lives. The presence of TOD is determined by the organs that depend heavily on a constant blood supply to function properly. Persistently elevated BP can predispose these organs to changes that result in secondary, often harmful, manifestations (Figure 7). Organs most likely to experience TOD include the kidneys, eyes, nervous system, and heart, particularly due to structural and functional modifications in the cardiovascular system (Figure 7) [93]. Acierno et al. [2] suggest that the presence of TOD indicates that therapeutic management should be initiated to prevent further progression and adverse outcomes. Target organs, with higher blood demands, often attempt to compensate for BP fluctuations, but in SAH patients, this compensation is insufficient, leading to specific and damaging lesions [87,93].

Cardiovascular TOD primarily result from direct damage caused by SAH [2]. Evidence indicates underlying heart diseases, such as hypertensive heart disease in dogs, may be present [93,103,104]. Thoracic radiographs are a valuable tool in assessing aortic remodelling due to SAH, as they can reveal vascular remodelling and enlargement [104,105] (Figure 7).

The ocular system can also be affected, with various studies identifying distinct symptoms and conditions (Figure 7) [2,106,107,108,109,110,111,112]. Hypertensive retinopathy and choroidopathy are the most commonly observed ocular conditions, with retinal detachment being the most frequent (Figure 7) [107,110,111]. A study by Cole et al. [111] found that about 16% of dogs showed signs of hypertensive retinopathy. Chalhoub and Palma [86] and Holt et al. [112] indicated these conditions result from the breakdown of the blood–retinal barrier, leading to fluid and protein leakage. Most ocular disorders caused by SAH are irreversible [48] and are more common in cats [93]. While BP management can help control the condition, it may not restore ocular health [107], with funduscopy recommended for assessment [110] (Figure 7).

Renal TOD occur due to the kidneys’ rich vascularisation, which allows for the detection of proteinuria and renal injury (Figure 7) [87,88,93]. After the initiation of antihypertensive therapy, it is expected that renal damage progression will be controlled, along with a reduction in proteinuria [1,88]. Glomerulosclerosis and hyperplastic arteriolosclerosis may prevent effective BP control (Figure 7) [113]. Given the risk of renal TOD, the IRIS [88] recommends sub-staging animals based on their BP, which guides diagnostic and therapeutic decisions. According to IRIS [88], “patients are sub-staged by systolic blood pressure according to the degree of target organ injury risk and whether evidence of target organ injury or complications is present” (Figure 7).

Neurological TOD, including hypertensive encephalopathy and white matter oedema, are also associated with SAH (Figure 7) [87,93]. These conditions are more common in cats [48,93,114]. Mathur et al. [115] found that antihypertensive management could help control these neurological disturbances. SAH-related neurological symptoms may include “ataxia, circling, stupor, coma, seizures, blindness, nystagmus, and behavioural and balance changes” (Figure 7) [93]. Additionally, evidence suggests the occurrence of haemorrhage, infarcts, vasogenic oedema, and cervical myelopathy due to ischaemia [116,117,118] (Figure 7).

## 9. Diagnosis and Devices of Measurement

The diagnosis of hypertension requires serial BP measurement [1], considering factors that may interfere with accuracy. To obtain reliable results, it is essential to follow a protocol that can be consistently replicated. Variability and potential unreliability in BP data are often due to changes in technique, patient position, and the operator’s experience. Situational SAH can be minimised by measuring BP in a quiet area after the animal has acclimatised, away from other animals, and before any procedures [2]. If possible, having the animal’s owner present and minimising restraint is beneficial, as BP and heart rate may be elevated when measurements are made without the owner [119]. BP values and risk for TOD are shown in Table 1.

Canine-friendly handling techniques facilitate patient management and help reduce stress, which is one of the main barriers to accurate BP measurement. A study by Navarro et al. [120] found that 86% of veterinary professionals use non-invasive BP measurement in cats. However, 4.1% of cases did not perform BP measurements, primarily due to anxiety, fear, and stress in animals. Discomfort during the procedure and difficulty in execution were also significant issues for 40% and 57% of professionals, respectively [120]. Approximately 90% of veterinary professionals apply techniques to reduce situational SAH, with the most common methods being quiet location (71.7%), minimal restraint (49.9%), measurement before any procedure (47.7%), avoiding other animals (34.6%), and allowing acclimatisation time (26.4%).

**Figure 8:**
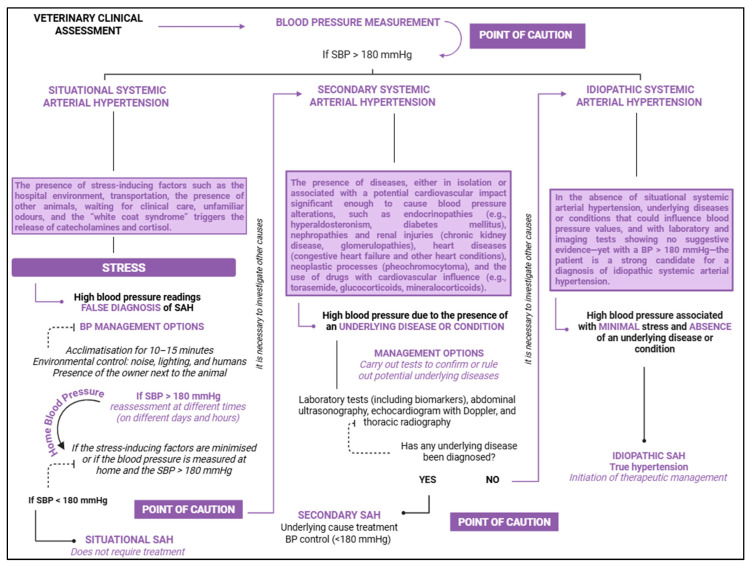
Flowchart to assist in the classification and diagnosis of types of systemic arterial hypertension, including definition criteria and monitoring. BioRender 2025 (https://www.biorender.com/ (accessed on 20 April 2025)). Created by the authors.

The operator’s experience significantly influences BP measurement accuracy. Operators should be qualified and experienced in patient handling and equipment use [2]. Gouni et al. [121] demonstrated that professionals with more training are more likely to obtain reliable and faster BP values. Proper cuff selection is also crucial for accurate measurements, with the cuff width being 30–40% of the circumference of the measurement site [2]. Smaller cuffs tend to overestimate BP, while larger cuffs underestimate it, affecting clinical decisions regarding SAH diagnosis [1]. This approach will guide the clinical approach to managing SAH. Prost [122] found that 80% of veterinary professionals do not recommend BP measurement in cats over 7 years old due to cost, stress, and time constraints, and when it is performed, it is usually in the presence of comorbidities. Figure 8 presents a flowchart outlining the types of SAH, starting from the diagnostic stage and including the steps required for an accurate diagnosis.

According to Acierno et al. [2], SAH cases can be diagnosed and classified into stages based on systolic BP values, which help direct clinical and emergency management strategies (Table 1). Reliable measurements are critical for this classification, with systolic BP being commonly used for diagnostic purposes [2,123]. Correct measurements are essential for therapeutic management decisions, as incorrect readings cannot form the basis for an SAH diagnosis [1]. Given the complications associated with TOD, the presence of lesions from a single BP measurement already suggests the need for antihypertensive management. However, subsequent confirmation with additional measurements is recommended [2]. Acierno et al. [2] suggest that animals showing signs of pre-hypertension or moderate TOD risk should have BP measurements every 4–8 weeks, while those with higher BP values and high TOD risk should be monitored every 1–2 weeks [2].

BP measurement can be performed using invasive and non-invasive methods, with the latter being more commonly used in routine practice [1]. Although the invasive method is the gold standard for BP measurement, the required infrastructure and operator experience can make it impractical for routine use. This method involves inserting a catheter into an artery connected to a device that measures BP [1,124]. The main challenges include the operator’s experience and the pain associated with the procedure, so it is typically restricted to anaesthesia or intensive care cases [1,124].

Non-invasive methods such as Doppler vascular method and oscillometry are more commonly used. The Doppler method involves complete arterial occlusion with a sphygmomanometer, and BP is measured as blood flow is restored during deflation. The oscillometric method, as described by Sousa et al. [1], “is based on occlusion and release of arterial flow, monitoring returning pulses, and emitting systolic, diastolic, and mean pressure values along with heart rate”. Studies comparing non-invasive methods have produced mixed results. Vachon et al. [124] compared oscillometric and Doppler methods with invasive BP measurements in medium to large breed dogs under anaesthesia. Oscillometry showed better correlation with invasive BP, particularly for mean and diastolic BP values, while Doppler was less effective. Stethoscopes can assist in BP measurement, especially for patients sensitive to noise. While Gill et al. [125] found no difference in BP values when using stethoscopes, the results were more precise. In contrast, a study by Uematsu et al. [126] showed that stethoscopes reduced BP readings in elderly cats (137 ± 17 mmHg vs. 125 ± 15 mmHg). For BP measurements, Acierno et al. [2] suggest “The first measurement should be discarded. A total of 5–7 consecutive consistent values should be recorded. In some patients, measured BP trends downward as the process continues. In these animals, measurements should continue until the decrease plateaus and then 5–7 consecutive consistent values should be recorded”.

Navarro et al. [120] found that nearly 70% of veterinary professionals prefer the Doppler vascular method due to its reliability. Taylor et al. [123] noted that oscillometry presents challenges, and in some cases, the device may fail to measure BP. Martel et al. [127] found a positive correlation between systolic BP values obtained by invasive and high-definition oscillometric devices in cats. Anjos et al. [128] compared oscillometry and Doppler in conscious healthy cats and observed significant differences in average values, though these differences were not clinically relevant. Nevertheless, both methods consistently showed higher BP values. Moreover, in 2025, Mantovani et al. [129] conducted a study aimed at comparing the accuracy of linear deflation oscillometry (LDO) and Doppler measurements against invasive BP monitoring in dogs under anaesthesia. The authors observed that Doppler tends to overestimate systolic BP values during hypotensive episodes. Additionally, they found that LDO is effective in detecting hypotension in anaesthetised dogs.

## 10. Therapeutic Management

**Figure 9:**
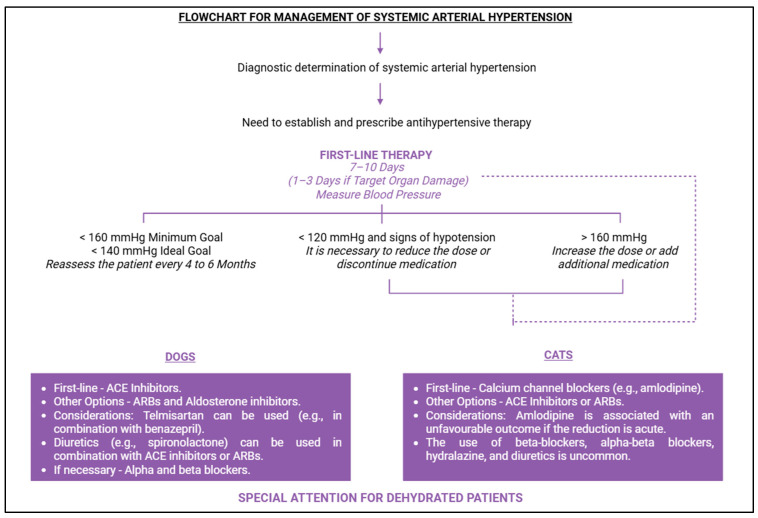
Flowchart for the implementation of therapeutic management for systemic arterial hypertension, including the most commonly used pharmacological classes by species. Adapted from Acierno et al. [2]. BioRender 2025 (https://www.biorender.com/ (accessed on 20 April 2025)). Created by the authors.

Antihypertensive therapy should be initiated once the presence of either idiopathic or secondary hypertension is confirmed, provided that BP values exceed the reference threshold after successive and accurate measurements. Yamato [93] identifies five key factors to consider when starting antihypertensive management: serially elevated BP, the presence of secondary diseases, evidence of TOD, impairment due to TOD, and the exclusion of situational hypertension. Timely clinical and therapeutic monitoring is essential for patients with secondary diseases to control the effects of elevated BP [2]. Once antihypertensive therapy begins, affected animals are unlikely to return to normotension and remain at risk for TOD [2]. Acierno et al. [2] emphasise that “the treatment of a patient’s hypertension should not be delayed until the underlying condition is controlled”. Additionally, it is crucial that pet owners are informed about their animal’s condition, its consequences, potential complications, and the need for ongoing treatment. Figure 9 summarises the strategies for the therapeutic management of SAH, including the medications used for both dogs and cats.

Antihypertensive therapy should be implemented alongside the treatment of any underlying conditions [2]. Early identification and the establishment of treatment strategies are necessary for optimal management. After initiating antihypertensive therapy, animals must be reassessed regularly to monitor BP values and determine whether the prescribed medication(s) and dosages are appropriate, or if adjustments are needed [1]. Like any clinical treatment, management must be individualised to suit each patient’s specific needs [1]. Acierno et al. [2] recommend gradual BP control and management to avoid complications from sudden reductions in BP.

From a pharmacological standpoint, the first-line treatment for dogs involves medications that target the RAS [2], such as ACE inhibitors, angiotensin II receptor blockers (ARBs), and aldosterone inhibitors [2,93], with ACE inhibitors being the most commonly recommended (Figure 9) [1]. ACE inhibitors are particularly beneficial for controlling blood volume, PVR, and maintaining proteinuria [93]. King et al. [130] demonstrated that benazepril effectively controls proteinuria. Mishina and Watanabe [131] showed that benazepril (2 mg/kg, q24h, for 2 weeks) effectively reduced BP, angiotensin II, and aldosterone levels, with values returning to baseline after discontinuation. Ames et al. [132] noted that benazepril or enalapril effectively block ACE activity, which is beneficial for BP control. Telmisartan has also been shown to control BP, with potential for combination therapy with benazepril (Figure 9). Fowler et al. [133] found that combining ACE inhibitors (mean dose 1.75 mg/kg, q24h) with telmisartan (mean dose 0.93 mg/kg, q24h) resulted in a 13 mmHg reduction in BP and a decrease in the protein/creatinine ratio in urine. However, LeCavalier et al. [134] found that using telmisartan alone did not alter BP values. Diuretics like spironolactone may be considered in combination with ACE inhibitors or ARBs [93]. Patients with specific conditions, such as adrenal tumours and aldosterone overproduction, may benefit from BP control using alpha or beta blockers (Figure 9) [93].

Regarding felines, the first-line pharmacological treatment typically involves the use of calcium channel blockers such as amlodipine (Figure 9) [93,135,136]. The administration of amlodipine can result in a blood pressure reduction of between 28 and 55 mmHg [17,136]. Huhtinen et al. [136] recommend a dose of 0.625 mg per cat if systolic blood pressure (SBP) is below 200 mmHg and 1.25 mg/kg if SBP exceeds 200 mmHg. Morita et al. [135] conducted a study to evaluate the effects of amlodipine in cats, involving 80 animals divided into three groups: amlodipine alone, amlodipine combined with telmisartan, and amlodipine combined with benazepril. Approximately 75% of the cats received amlodipine as monotherapy, with a mean daily dose of 0.41 ± 0.51 mg/kg. The authors observed reductions in systolic, diastolic, and mean BP across all treatment groups, with the most significant decrease seen in the amlodipine + telmisartan group [135]. Furthermore, the reduction in systolic BP following amlodipine administration was greater in cats with an SBP below 140 mmHg [135]. Additionally, cats with an SBP ≥ 140 mmHg demonstrated higher survival rates compared to those with an SBP < 140 mmHg after receiving amlodipine [135]. According to Acierno et al. [2], doses exceeding 2.5 mg per cat are rarely necessary. Sent et al. [137] suggest that amlodipine may be used in combination with either ACE inhibitors or ARBs, although ACE inhibitors are not recommended as first-line therapy [2]. Caution is advised when prescribing these medications to dehydrated cats, particularly those affecting the RAS (Figure 9) [2]. The use of beta-blockers, alpha-beta blockers, hydralazine, and diuretics is uncommon [2,93].

## 11. Conclusions

The importance of performing BP measurements as a standard clinical practice in veterinary settings is clear. Hypertension is defined by a persistent elevation in BP values and can be classified into different types: one caused by catecholamine excess, another linked to underlying diseases, and true hypertension, where other causes have been excluded. Hypertension is a condition with variable progression, impact, and severity, and is often described as a silent process. Therefore, serial measurements are essential, taking into account variables that may influence the values, such as stress, size, environment, and the method of measurement. It is crucial to understand that hypertension carries the potential risk of TOD, which can affect different organs, with the consequences varying depending on the organ involved. Early and accurate identification of hypertension enables the initiation of pharmacological therapy to prevent the condition from worsening and minimise the onset of organ damage. Veterinary professionals must be aware of the importance of BP measurement, which should be performed at regular intervals. This approach will allow for a timely diagnosis of hypertension, guiding the implementation of clinical and/or emergency pharmacological therapy. Such measures will ultimately impact the patient’s life expectancy and quality of life, as well as that of the owner.

## Figures and Tables

**Table 1:** Systolic blood pressure values and risk for target organ damage for companion animals [2].

Systolic Blood Pressure	Classification	Risk for TOD
<140 mmHg	normotensive	minimal
140–159 mmHg	prehypertensive	low
160–179 mmHg	hypertensive	moderate
≥180 mmHg	severely hypertensive	high

## Abbreviations

The following abbreviations are used in this manuscript:

ACE	Angiotensin-converting enzyme
ACVIM	American College Veterinary Internal Medicine
ARBs	Angiotensin II receptor blockers
BP	Blood pressure
CKD	Chronic kidney disease
CO	Cardiac output
IRIS	International Renal Interest Society
mmHg	millimetres of mercury
PNS	Parasympathetic nervous system
PVR	Peripheric vascular resistance
RAAS	Renin–angiotensin–aldosterone system
RAS	Renin–angiotensin system
SAH	Systemic arterial hypertension
SNS	Sympathetic nervous system
TOD	Target organ damage
VR	Venous return
